# Decision analysis framework for predicting no-shows to appointments using machine learning algorithms

**DOI:** 10.1186/s12913-023-10418-6

**Published:** 2024-01-05

**Authors:** Carolina Deina, Flavio S. Fogliatto, Giovani J. C. da Silveira, Michel J. Anzanello

**Affiliations:** 1 Department of Industrial Engineering, Federal University of Rio Grande do Sul, Av. Osvaldo Aranha, 99, 5° Andar, Porto Alegre, 90035-190 Brazil; 2 Haskayne School of Business, University of Calgary, 2500 University Dr NW, Calgary, AB T2N 1N4 Canada

**Keywords:** Missed appointments, Healthcare environments, Imbalanced dataset, Classification algorithms, Resampling techniques, Machine learning

## Abstract

**Background:**

No-show to medical appointments has significant adverse effects on healthcare systems and their clients. Using machine learning to predict no-shows allows managers to implement strategies such as overbooking and reminders targeting patients most likely to miss appointments, optimizing the use of resources.

**Methods:**

In this study, we proposed a detailed analytical framework for predicting no-shows while addressing imbalanced datasets. The framework includes a novel use of *z*-fold cross-validation performed twice during the modeling process to improve model robustness and generalization. We also introduce Symbolic Regression (SR) as a classification algorithm and Instance Hardness Threshold (IHT) as a resampling technique and compared their performance with that of other classification algorithms, such as K-Nearest Neighbors (KNN) and Support Vector Machine (SVM), and resampling techniques, such as Random under Sampling (RUS), Synthetic Minority Oversampling Technique (SMOTE) and NearMiss-1. We validated the framework using two attendance datasets from Brazilian hospitals with no-show rates of 6.65% and 19.03%.

**Results:**

From the academic perspective, our study is the first to propose using SR and IHT to predict the no-show of patients. Our findings indicate that SR and IHT presented superior performances compared to other techniques, particularly IHT, which excelled when combined with all classification algorithms and led to low variability in performance metrics results. Our results also outperformed sensitivity outcomes reported in the literature, with values above 0.94 for both datasets.

**Conclusion:**

This is the first study to use SR and IHT methods to predict patient no-shows and the first to propose performing *z*-fold cross-validation twice. Our study highlights the importance of avoiding relying on few validation runs for imbalanced datasets as it may lead to biased results and inadequate analysis of the generalization and stability of the models obtained during the training stage.

**Supplementary Information:**

The online version contains supplementary material available at 10.1186/s12913-023-10418-6.

## Introduction

No-shows of patients or late cancellations of medical appointments, which do not allow the use of the assigned time interval for other purposes, are reported as common events in different medical practices [1–3]. Their consequences impact health systems and clients [4], as they imply a lack of care for two patients: one who did not attend the appointment and another who could not schedule an appointment at the assigned timeslot [1]. On the healthcare provider side, patient no-show leads to inefficient use of resources, manifested by increased costs and employee idleness, and limited access to the service by other patients [5, 6]. On the patient side, no-shows impact the continuity of care, worsening clinical outcomes and increasing dissatisfaction with longer waiting lists [1, 7].

Some strategies have been conceived to mitigate the no-show problem, including appointment reminders to patients through calls and text messages [6, 8]. Although promoting some behavior change, the effect of such practices can be limited [9]. Financial penalties have also been suggested [7, 10]; however, that may not be appropriate, as it may constrain access to care by many in the population [11]. Another practice to reduce revenue losses associated with nonattendance is overbooking [8, 10]; however, when patients attend appointments, there will be scheduling collisions and extended waiting lists, which may negatively affect patients [3].

Mathematical modeling of patients’ no-show probabilities, as other domains, is a crucial asset for decision-makers striving to implement more effective and proactive countermeasures. This approach diminishes the subjectivity surrounding the event, guiding more informed plans and perceptions to alleviate the problem [8, 10, 12]. Patients’ attendance is an event with two possible outcomes (0 – show or 1 – no-show), which may be predicted using classification algorithms.

The performance of no-show modeling may benefit from the testing of methods not yet explored in the literature. To the best of our knowledge, no studies used the Symbolic Regression (SR) algorithm for prediction or the Instance Hardness Threshold (IHT) technique for data balancing. SR is an algorithm that does not initially have a pre-specified mathematical structure, which is inferred from the data by manipulating mathematical expressions during the algorithm’s evolution via genetic programming. One of the main advantages of SR is observable in the algorithm specification step, as the algorithm has a higher chance of finding a mathematical structure that fits the data well due to its ability to explore a wide range of possible models [13]. The IHT technique is judicious in balancing the data using a hardness property that acts as a filter to exclude data that may be outliers, noise, or that overlap the sample space of the minority class [14, 15].

In this paper, we analyze the problem of no-show to medical appointments. Our two research objectives are:

- To propose an analytical framework that utilizes machine learning algorithms for predicting no-shows to appointments, while effectively addressing the challenge of imbalanced datasets. Our framework is intended to be adaptable to medical no-shows of any nature.
- Test classification and balancing methods not yet explored in the no-show prediction literature (i.e., SR and IHT) and compare their performance with that of traditional classification algorithms, e.g., K-Nearest Neighbors (KNN) and Support Vector Machine (SVM), and resampling techniques, e.g., Random under Sampling (RUS), Synthetic Minority Oversampling Technique (SMOTE) and NearMiss-1.

We analyze two datasets of attendance at hospitals in Brazil. The first is unpublished data on out-patient computed tomography (CT) scheduling obtained from the appointment scheduling management system of the Department of Radiology of a public tertiary teaching hospital in southern Brazil, totaling 8,371 appointments (6.65% no-shows). The second is a public dataset from a healthcare provider system in southeastern Brazil acquired from the data science competition platform Kaggle, containing 7,717 appointments (19.03% no-shows) [16]. The use of two databases aims to better explore the analytical algorithm and techniques, strengthening the results. Prediction results for the datasets are not directly comparable since they included different sets of predictors.

The contributions of our work can be summarized regarding its theoretical and practical aspects, as follows.

As for the state-of-the-art, we propose an analytical framework for predicting no-shows to appointments. We address the challenge of imbalanced datasets by proposing a novel use of z-fold cross-validation, which is performed twice during the modeling process. The approach enhances the robustness and generalization of the predictive models. It also allows a more comprehensive assessment of performance metrics by determining their centrality and dispersion statistics and minimizes the possibility of bias in the composition of the calibration and validation sets. Additionally, we explore methods not yet used to predict no-shows in healthcare centers (namely, SR and IHT), and novel combinations of classification algorithms and data balancing techniques.

As for practical aspects, estimating no-shows within an integrated e-Health system can significantly enhance healthcare quality. It enables transparent access to patient information, empowering managers to devise targeted intervention strategies for patients who most likely not to attend appointments. Ying et al. [17] and Huang & Hanauer [18]. Accurate no-show forecasts may also improve the performance of well-known strategies to reduce the impacts of no-shows on the system’s performance, such as scheduling overbooking [3]. For example, a study by Huang & Hanauer [18] compared two overbooking strategies, with and without the use of no-show predictions. The authors reported a reduction of at least 6% in patients’ waiting times, 27% in employees’ overtime hours, and 3% in total costs when using no-show predictions.

## Background

Patient no-shows in medical appointments are a common event across various specialties [19], leading to ongoing investigation of mathematical approaches to address nonattendance issues and support decision-making for service managers. These approaches involve identifying predictors influencing no-shows and developing predictive models [3, 20, 21].

Predictors of nonattendance are categorized into three main groups: (a) sociodemographic, e.g., age [5], gender [6], ethnicity [22], marital status [8], education [23], language [24], distance to the consultation site [25], and median household income [24]; (b) scheduling characteristics, e.g., appointment day [26], hour of the day [2], month of the appointment [3], season of the year [19], days elapsed between scheduling and appointment [4], medical specialty [27], multiple appointments on the same day [7], and examination’s risk modality [10]; and (c) history of nonattendance, e.g., prior no-show [28], and past appointment history [29].

However, the generalizability of results across studies is challenging due to the case-specific nature of nonattendance influenced by internal and external factors unique to each medical service. For instance, gender’s significance as a no-show predictor varies, with some studies reporting higher rates in males [5] and others in females [30]. Despite this variability, context-specific studies help managers devise compensating strategies. For instance, Glover et al. [24] associated no-shows with transport barriers for low-income patients, implementing strategies such as directing patients to geographically accessible clinics, creating partnerships with transport managers, and proposing a free transportation program for vulnerable patients.

Several no-show prediction models have been proposed, with logistic regression and its variants being predominant, justified by their traditional use in modeling binary responses. However, recent works increasingly explore machine learning models such as Artificial Neural Network and Gradient Boosting. Notably, only 27 of the 62 works identified on the subject compared classification algorithm performance, and logistic regression was not pointed as the best-performing model in any case. Comparing algorithm performance is crucial for reliable results [3, 28, 31–41].

A novel approach, Symbolic Regression (SR), remains unexplored in no-show prediction studies. Unlike traditional regression algorithms, SR explores various mathematical associations between predictors, both linear and nonlinear. Employing genetic programming, SR evolves through a tree structure, manipulating mathematical expressions to discover functions describing the dataset [13]. Examples of SR applications in prediction may be found in Yamashita et al. [42] and Chaabene and Nehdi [43].

Feature selection is vital for classification algorithms, reducing redundant variables and improving predictive capability. Filter, wrapper, and embedded feature selection approaches are reported in the literature. Filter methods assign scores to features using statistical metrics, independent of the classification algorithm, making them computationally fast. Wrapper methods, while computationally demanding and prone to overfitting, often yield the best performance by selecting features within the classification algorithm. Embedded methods, rooted in the classification algorithm building process, are less computationally demanding and prone to overfitting [44].

The imbalance between classes in no-show modeling poses challenges to machine learning algorithms, favoring naive approaches and generating biased results as dataset imbalance increases. Resampling techniques, such as minority class oversampling, majority class undersampling, or combinations of both, address this issue [35]. Studies in Supplementary Table S1 show that the no-show rate is generally lower than the attendance rate in most medical specialties, but class imbalance impacts prediction, with sensitivity results below 47% in some cases [28, 40, 41, 45–47].

In classification problems, distinguishing between type I (false positives) and type II (false negatives) errors is crucial. Sensitivity, measuring the model’s ability to predict positive class occurrences (no-shows), is vital for performance assessment. In healthcare, models are considered more sensitive if they correctly identify patients who will not attend appointments, as no-shows incur higher costs and resource waste. Resampling techniques improve sensitivity, with studies favoring majority class undersampling, particularly using Random Under-Sampling (RUS) [35]. One undersampling technique not explored in the no-show modeling literature is Instance Hardness Threshold (IHT). IHT uses hardness measurements to filter out instances that may be outliers, noise, or overlap the minority class sample space [14, 15].

Finally, a cross-validation process is essential for assessing predictive model capability [48, 49]. Cross-validation ensures accurate parameter settings for machine learning algorithms, minimizing overfitting during the training phase [37, 38, 40]. Implementing proportional class sampling (Stratification by class) when splitting the dataset into cross-validation folds enhances result reliability [37, 38, 40].

### Resampling techniques

In classification problems, the objective is to minimize prediction errors by assigning class labels to observations. However, in the context of no-show prediction, the minority class is crucial, and minimizing errors in that class is essential [28, 32]. Resampling techniques address class imbalance, and in our application, we compared four techniques using the Imbalanced-learn Python toolbox [50]: Synthetic Minority Oversampling Technique (SMOTE), Random UnderSampling (RUS), NearMiss (NM), and Instance Hardness Threshold (IHT).

SMOTE, an oversampling technique, generates synthetic observations for the minority class. In each iteration, an observation $x$ is randomly selected from the minority class, its $k$ nearest neighbors are identified, and one of them is randomly selected (say ${k}^{*}$). The Euclidean distance between $x$ and ${k}^{*}$ [denoted by $d\left(x,{k}^{*}\right)$] is calculated; the result is multiplied by a random value between 0 and 1 [denoted by $rand\left(0, 1\right)$] and added to $x$ to generate the synthetic observation $x{\prime}$, i.e. ,

1 $${x}{\prime}=x+\left[rand\left(0, 1\right)\times d\left(x,{k}^{*}\right)\right]$$

RUS, an undersampling technique, randomly removes observations from the majority class until it matches the minority class size, potentially losing valuable information [35]. NM, another undersampling approach, balances classes based on the Euclidean distance. It removes observations from the majority class with the shortest distances to the minority class, aiming to increase class separation [51].

IHT is an undersampling technique that eliminates observations of the majority class based on a hardness property. Every dataset observation (or instance) may be characterized by its respective probability of being misclassified, i.e., the instance hardness value. An outlier, for example, is expected to have a large hardness value, and the learning algorithm will probably overfit the model to correctly classify the observation. The objective of machine learning algorithms is to maximize $p\left(h|t\right)$, where $h:X\to Y$ represents a function that maps the observation vector $X$ onto the label vector $Y$, and $t=\{\left({x}_{i}, {y}_{i}\right):{x}_{i}\in X \bigwedge {y}_{i}\in Y\}$ represents the training set. The Instance Hardness (*IH*) is obtained from the decomposition of $p\left(h|t\right)$ using Bayes’ theorem (Eq. 2) [14, 15].

2 $$p\left(h|t\right)=\frac{p\left(t|h\right) p\left(h\right)}{p\left(t\right)} =\frac{{\prod }_{i=1}^{\left|t\right|}p\left({x}_{i}, {y}_{i}|h\right) p\left(h\right)}{p\left(t\right)}= \frac{{\prod }_{i=1}^{\left|t\right|}p\left({y}_{i}|{x}_{i}, h\right) p\left({x}_{i}|h\right) p\left(h\right)}{p\left(t\right)}$$

For each observation ${x}_{i}$ of the training set, the probability $p\left({y}_{i}{|x}_{i}, h\right)$ of function $h$ correctly assigning a label ${y}_{i}$ to the observation is calculated. The definition of Instance Hardness (*IH*) with respect to $h$ is represented by:

3 $$I{H}_{h}\left({x}_{i}{, y}_{i}\right)=1-p\left({y}_{i}{|x}_{i}, h\right)$$

The largest the $p\left({y}_{i}{|x}_{i}, h\right)$, the smaller the instance hardness value; in opposition, large probability values indicate instances for which classification assertiveness is lower. In practice, function $h$ is determined using some learning algorithm, e.g., in Python’s *Imbalanced-learn* the default is the random forest algorithm [52]. More details about IHT can be found in Smith et al. [14], where the empirical evaluation of the IHT technique in other application domains showed significant improvements in predictive accuracy compared to other classification methods, particularly in situations with severe class imbalance.

### Prediction models

In this study, we employed the KNN, SVM, and SR supervised algorithms for predicting patient no-shows. The KNN algorithm determines the class of a new observation by majority voting based on the *k* nearest observations. Cross-validation is crucial for determining the optimal value of *k*, avoiding model overfitting or underfitting [53].

The SVM algorithm establishes a decision boundary (hyperplane) between classes, aiming to maximize the margin of separation. It uses a regularization parameter (*C*) to balance the penalty for misclassifications, influencing the trade-off between bias and variance [53, 54]. SVM can perform nonlinear classification using the kernel trick, mapping observations into a higher-dimensional feature space.

SR, a nonlinear regression technique, evolves through Genetic Programming (GP) by combining user-specified mathematical functions. It lacks a pre-defined model structure, with the best-fit model evolving through GP’s crossovers and mutations [55, 56]. SR’s candidate solutions are represented as trees, where top nodes indicate mathematical functions connecting expressions in bottom nodes. Further details on SR and GP are available in Poli et al. [56] and Koza [13].

## Method

**Fig. 1:**
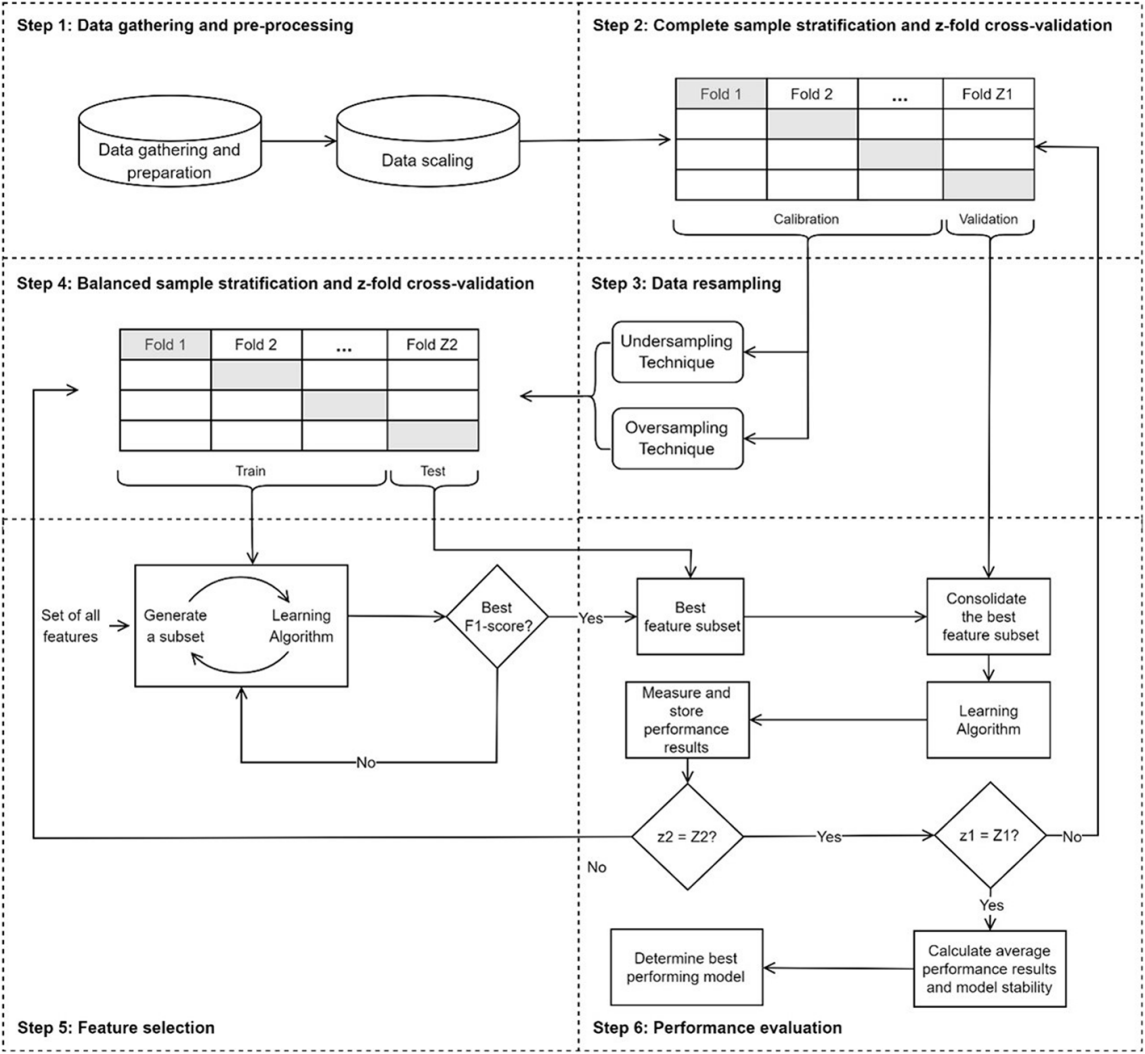
Outline of the proposed framework

To provide information to help clinics adopt strategies to minimize problems related with no-shows, we propose a six-step predictive framework (Fig. 1). In what follows, steps are detailed in the context of our application. We review resampling techniques, predictive modeling approaches, and performance metrics, emphasizing those that are new in the context of no-show prediction.

In our application, two datasets are analyzed. Dataset 1 is an unpublished database with information from the appointment system of the Department of Radiology of Hospital de Clinicas de Porto Alegre, a public tertiary teaching hospital located in Rio Grande do Sul State, Brazil. Dataset 2 is an open-access database consolidating information from the public healthcare system of Espirito Santo State, Brazil. As the databases have different characteristics, both are specific cases, not allowing direct comparisons of their results. The intentional incorporation of datasets with distinct characteristics was a deliberate choice to explicitly test the robustness of the proposed model in various clinical contexts. This decision is based on the need to address the inherent complexity and diversity of health data, ensuring the adaptability of the structure of our proposed model to medical no-shows of any nature.

In the first step, data gathering and pre-processing take place. We adopted an approach that combined graphical visualization and descriptive statistics to identify and assess outliers that were beyond the expected range. Given the presence of a few missing data points and outliers, we chose to carefully remove these elements. After pre-processing, both datasets were scaled such that all continuous variables’ outcomes were in the [0,1] interval. Max–min scaling is displayed in Eq. (4), in which $X$ represents the variable outcome, ${X}_{max}$ is the largest outcome in the sample and ${X}_{min}$ is the smallest outcome.

4 $$Scaling =\left(X - Xmin\right)/(Xmax - Xmin)$$

In the second step, we divide the complete dataset to obtain calibration and validation portions using the $z$-fold cross-validation technique. Since the same technique is applied in steps 2 and 4 on different versions of the dataset (complete and balanced), we denote the total number of folds in this step by ${Z}_{1}$ (${z}_{1}=1,\dots ,{Z}_{1}$). In our application, ${Z}_{1}$ was set to 10, such that nine folds were used to obtain the calibration portion, and one fold was used for validation since the model can be more generalizable using larger training sets [3, 57]. As the number of observations in each class (show and no-show) is different in the dataset, each fold was obtained to reflect the class proportions of the complete sample. That was guaranteed using a stratified randomized sampling technique.

In step 3, we apply resampling techniques to the calibration portion of the dataset. In our application, four techniques were tested: SMOTE, RUS, NM, and IHT; they were presented in “Background” section. The selection of RUS and SMOTE was guided by their extensive utilization in the no-show literature [26, 35, 58–60]. Abushaaban & Agaoglu [59] influenced our choice of the NM technique, given its relatively unexplored nature in the literature. Importantly, we intentionally incorporated the IHT technique with a specific emphasis on its novel application, as it has not been employed in previous no-show prediction studies.

In step 4, the balanced calibration portion of the dataset is divided into train and test portions. We once again use the $z$-fold cross-validation technique to randomly generate the partitions; the total number of folds in this step is denoted by ${Z}_{2}$ (${z}_{2}=1,\dots ,{Z}_{2}$). In our application, ${Z}_{2}$ was set to 10.

Feature selection takes place in step 5, using a wrapper method, which typically yields superior performance compared to other methods recognized in the literature (e.g., filter or embedded) [31]. Despite being computationally more intensive than these alternatives, our choice is justified by the type of dataset typically analyzed in no-show prediction problems, characterized by a small ratio of variables to observations and high imbalance between classes. In a wrapper method, feature selection is run on the machine learning algorithm being tested on the dataset. Using a greedy search approach, it tests all possible feature combinations and selects the one giving the best performance with respect to one or more evaluation criteria. In classification problems, typical criteria are accuracy, precision, recall, and F1-score. More details on wrapper methods are available in Kohavi and John [61] and Bolón-Canedo et al. [52].

In our application, we aimed to maximize the F1-score criterion, which was chosen to evaluate the performance of candidate feature subsets. Three supervised classification algorithms were trained: KNN, SVM, and SR; they were presented in “Prediction models” section, with special emphasis on SR, which was not used in previous no-show prediction studies. We implemented KNN and SVM using the open-source Python toolbox *scikit-learn* [62]. In the existing literature on no-show predictions, authors employing KNN and SVM as predictors did not report the parameters used.

For KNN, the best value of $k$ was determined by evaluating a range of possibilities (1, 3, 5, 7, 9, 11, 13, 15). While Uddin et al. [63] and Saini et al. [64] reported that the best values for $k$ generally fall between 1 and 9, Batista & Silva [65] work suggests that values for $k$ above 15 may lead to overfitting.

For SVM, we evaluated two of the most popular kernel functions [sigmoid and radial basis function (RBF)]. Both functions are suitable for handling problems where data is not linearly separable. We opted for the sigmoid kernel due to its suitability in scenarios with a small number of features, given its relatively simpler computational nature. On the other hand, the RBF kernel is popularly used as a default choice and has consistently demonstrated superior performance compared to other functions in the literature [66, 67]. We determined the best value for $C$ evaluating the following possibilities: 1, 50, 100, 150, 200, 250, 300. The decision to limit our investigation to these parameters stemmed from the computational complexity and time costs associated with exploring a broader parameter range.

We implemented the SR via genetic programming (SR/GP) algorithm using the open-source Python toolbox *gplearn* [68]. For SR, parameters were set as follows: mathematical functions = addition, subtraction, multiplication, division, square root, log, and absolute value; population size = 500 individuals; number of generations = 50; crossover probability = 90%; mutation probability = 1%. The parameters were set based on the gplearn library documentation. The chosen mathematical functions form a diverse set, enabling the algorithm to explore a wide range of mathematical operations. A larger population size (500 individuals) allows for a more diverse set of candidate solutions, potentially covering a broader search space. The number of generations (50) determines the iterations of evolving solutions, attaining a balance between exploration and exploitation of the search space. A high crossover probability (90%) means a higher likelihood of exchanging genetic material between individuals, facilitating exploration and sharing of promising solutions. The low mutation probability (1%) ensures that the search process does not overly disrupt potentially good solutions, focusing more on exploiting the current population.

The feature selection step was performed only on the calibration portion of the dataset (step 5). In step 6, the best feature subset and parameter configuration were determined and tested on the validation portion of the dataset for each learning algorithm. No class balancing is done in the validation portion since the objective is to test the performance of the classification model in a real-life situation. Performance results are measured and stored. Once all folds generated in steps 2 and 4 are run using our proposed framework, we check the stability of the prediction models by calculating the standard deviations and determine the model with the best overall performance using averages of the chosen performance metrics. The following performance metrics are used: accuracy, positive and negative predictive values (PPV and NPV, respectively), sensitivity, specificity, F1-Score, and AUC. All analyses were programmed in Python.

### Performance metrics

To assess the performance of the prediction models tested in this study, we treated the no-show class as positive (or class 1) and the show class as negative (or class 0). Therefore, possible classification outcomes are: True Positive (TP), i.e., no-show cases are correctly classified; False Positive (FP), i.e., show cases that are classified as no-show; True Negative (TN), i.e., show cases correctly classified; and False Negative (FN), i.e., no-show cases classified as show. By designating no-shows as the positive class, our aim was to prioritize the accurate identification of these cases. It aligns with the practical goal of minimizing false negatives, as these misclassifications could potentially lead to missed opportunities for preventive actions or interventions [28, 31]. Classification outcomes were used to calculate the following performance metrics [39, 46]:

5 $$\mathrm{Accuracy}=(TP+TN) /(TP+ TN + FP + FN)$$

6 $$\mathrm{Positive}\;\mathrm{predictive}\;\mathrm{value}\;\left(\mathrm{PPV}\right)=TP/(TP+FP)$$

7 $$\mathrm{Negative}\;\mathrm{predictive}\;\mathrm{value}\;(\mathrm{NPV})=TN/(TN+FN)$$

8 $$\mathrm{Sensitivity}= TP /(TP+ FN)$$

9 $$\mathrm{Specificity}= TN /(TN+ FP)$$

10 $$\mathrm F1\;\mathrm{Score}=2\times\frac{PPV\times Sensitivity}{PPV+Sensitivity}$$

In addition to the metrics above, we used the Area Under the Receiver Operating Characteristic Curve (AUC) for model performance evaluation. AUC outcomes range between 0 and 1, such that larger values correspond to more accurate classifications. A classifier yielding an AUC value of 0.5 is non-informative, giving the same results as randomly assigning classes to observations [3].

## Datasets

Dataset 1 has appointment records of patients who visited the hospital to perform CT scans between January 1 and December 31, 2017, totaling 12,387 appointments. Irrelevant (repeated and missing observations, outliers, etc.) data was discarded in pre-processing. The final dataset had 8,371 observations including 7,814 (93.35%) shows and 557 (6.65%) no-shows. To predict no-show events, the following 16 independent variables were considered: (a) sociodemographic information: age, gender, ethnicity, marital status, education level, distance to the clinic; (b) appointment information: day of the month, day of the week, scheduling shift, month of the year, season of the year, waiting days (i.e., time interval from scheduling to appointment), cancer history; (c) historical predictors of nonattendance: previous appointments (i.e., number of appointments scheduled in the previous year), previous no-shows (i.e., number of appointments with no-show in the previous year), number of exams scheduled in the previous year, number of exams with no-show in the previous year.

Dataset 2 was acquired from the Kaggle platform, which stores consultation records of patients that visited hospitals in different cities and neighborhoods of the Espirito Santo State (Brazil). The open access data were collected between April 29 and June 8, 2016, totaling 110,528 observations. We analyzed the largest data subset containing records of consultations in healthcare units of Jardim Camburi (a neighborhood of Vitoria, the capital city), totaling 7,717 observations. Pre-processing of data reduced the sample to 7,413 observations after removing inconsistent and incomplete records. The final dataset consisted of 6,002 (80.97%) shows and 1,411 (19.03%) no-shows. The following 12 independent variables were considered: (a) sociodemographic information: age, gender, governmental aid, handicap, alcoholism, hypertension, diabetes, SMS_received; and (b) appointment information: patient ID and appointment ID, comprised of scheduling date, appointment date, and appointment status (show or no-show). The information in variable ‘appointment date’ was deployed into three other variables: day of the month, day of the week, and month of the year. Four new variables were created using the information in (b); they were calculated for each appointment ID and are as follows: (i) ‘waiting days’, i.e., the number of days elapsed between the scheduling date and the appointment date; (ii) ‘previous appointments’, i.e., the total number of appointments scheduled prior to the present one, (iii) ‘no-shows in previous appointments’, i.e., the percentage of no-shows in appointments prior to the present one; and (iv) ‘number of days since previous appointment’, i.e., the number of days elapsed since the previous appointment. Once the new variables were created using the information in (b), the original variables were discarded, except for ‘appointment date’ and its deployed variables.

## Results

The processing of Dataset 1 following the framework steps in Fig. 1 led to the results reported in Tables 1 (test set) and 2 (validation set). Considering the cross-validations in steps 2 and 4, results presented are average values over 100 data replicates, with standard deviations estimated over 100 performance metrics results. The best results for each metric are marked in bold.

**Table 1:** Average predictive performance and standard deviations obtained from 100 replicates of dataset 1’s test portion

Resampling technique	Classification algorithm	Performance Metrics – mean (SD)						
		**AUC**	**Sensitivity**	**Specificity**	**NPV**	**PPV**	**F1_score**	**Accuracy**
SMOTE	KNN	0.8741 (0.081)	0.8783 (0.202)	0.8698 (0.047)	0.9044 (0.109)	0.8799 (0.039)	0.8571 (0.144)	0.8741 (0.081)
	SVM	0.6168 (0.042)	0.9273 (0.011)	0.3063 (0.083)	0.8017 (0.043)	0.5737 (0.033)	0.7083 (0.024)	0.6168 (0.042)
	SR	0.7055 (0.102)	0.5034 (0.250)	**0.9076 (0.186)**	0.6734 (0.109)	**0.9056 (0.147)**	0.5951 (0.210)	0.7055 (0.102)
RUS	KNN	0.5940 (0.053)	0.7183 (0.090)	0.4697 (0.076)	0.6312 (0.078)	0.5752 (0.043)	0.6372 (0.056)	0.5941 (0.053)
	SVM	0.6182 (0.036)	0.9281 (0.047)	0.3083 (0.065)	0.8194 (0.093)	0.5736 (0.024)	0.7085 (0.026)	0.6182 (0.036)
	SR	0.5644 (0.063)	0.5090 (0.416)	0.6198 (0.325)	0.6599 (0.172)	0.5231 (0.226)	0.4283 (0.294)	0.5642 (0.064)
NM	KNN	0.7509 (0.067)	0.7052 (0.059)	0.7966 (0.137)	0.7285 (0.046)	0.7957 (0.122)	0.7413 (0.058)	0.7509 (0.067)
	SVM	0.7111 (0.129)	0.7802 (0.087)	0.6420 (0.304)	0.6633 (0.232)	0.7320 (0.162)	0.7394 (0.079)	0.7109 (0.130)
	SR	0.7492 (0.095)	0.7256 (0.131)	0.7729 (0.203)	0.7355(0.110)	0.7981 (0.151)	0.7430 (0.093)	0.7492 (0.095)
IHT	KNN	**0.9087(0.032)**	0.9122 (0.052)	0.9052 (0.051)	0.9460 (0.030)	0.8586 (0.068)	**0.8822(0.041)**	**0.9079(0.034)**
	SVM	0.9017 (0.027)	0.9447 (0.042)	0.8588 (0.063)	0.9643 (0.024)	0.8074 (0.075)	0.8675 (0.035)	0.8909 (0.034)
	SR	0.9058 (0.032)	**0.9582(0.040)**	0.8533 (0.067)	**0.9728(0.025)**	0.8048 (0.076)	0.8720 (0.041)	0.8927 (0.038)

**Table 2:** Average predictive performance and standard deviations obtained from 100 replicates of dataset 1’s validation portion

**Resampling technique**	**Classification algorithm**	**Performance Metrics – mean (SD)**						
		**AUC**	**Sensitivity**	**Specificity**	**NPV**	**PPV**	**F1_score**	**Accuracy**
SMOTE	KNN	0.5321 (0.031)	0.1905 (0.080)	0.8736 (0.046)	0.9380 (0.004)	0.0912 (0.042)	0.1217(0.053)	0.8282 (0.039)
	SVM	0.6193 (0.029)	0.9320 (0.038)	0.3067 (0.080)	0.9847 (0.006)	0.0883 (0.008)	0.1611 (0.013)	0.3483 (0.073)
	SR	0.5141 (0.033)	0.1195 (0.253)	**0.9087 (0.191)**	0.9378 (0.011)	0.0399 (0.070)	0.0399 (0.060)	**0.8562 (0.161)**
RUS	KNN	0.6132 (0.036)	0.7432 (0.075)	0.4833 (0.065)	0.9636 (0.009)	**0.0938 (0.011)**	0.1664 (0.018)	0.5006 (0.059)
	SVM	0.6177 (0.031)	0.9366 (0.039)	0.2988 (0.079)	0.9854 (0.007)	0.0878 (0.008)	0.1603 (0.014)	0.3413 (0.072)
	SR	0.5693 (0.062)	0.5099 (0.422)	0.6286 (0.316)	0.9603 (0.025)	0.0792 (0.034)	0.1175 (0.063)	0.6208 (0.267)
NM	KNN	0.5211 (0.030)	0.7525 (0.061)	0.2898 (0.039)	0.9422 (0.013)	0.0702 (0.005)	0.1285 (0.009)	0.3206 (0.035)
	SVM	0.5146 (0.027)	0.8266 (0.076)	0.2025 (0.086)	0.8935 (0.232)	0.0690 (0.004)	0.1273 (0.007)	0.2441 (0.077)
	SR	0.5045 (0.030)	0.7367 (0.128)	0.2722 (0.119)	0.9355 (0.016)	0.0673 (0.005)	0.1231 (0.009)	0.3031 (0.103)
IHT	KNN	**0.6302 (0.037)**	0.8998 (0.051)	0.3606 (0.072)	0.9804 (0.009)	0.0921 (0.011)	**0.1670 (0.018)**	0.3964 (0.067)
	SVM	0.6204 (0.027)	0.9463 (0.044)	0.2945 (0.084)	0.9879 (0.006)	0.0881 (0.008)	0.1611 (0.013)	0.3378 (0.076)
	SR	0.6175 (0.035)	**0.9537 (0.042)**	0.2813 (0.075)	**0.9886 (0.009)**	0.0872 (0.009)	0.1596 (0.015)	0.3260 (0.069)

In classification problems, it is not possible to define a priori that all errors are equivalent since, depending on the case, type I (i.e., false positives = show events predicted as no-show) or type II errors (i.e., false negative = no-show events predicted as show) may have different impacts on the system. In healthcare centers, for example, prediction models are deemed more sensitive if they can identify patients who will not attend appointments correctly [28, 32]. As no-shows lead to higher costs and waste of resources, the cost of a false negative is usually higher than that of a false positive, so it is important that false negatives are minimized [28, 31, 60]. In situations of class imbalance, sensitivity and AUC often take precedence as they provide critical insights into a model’s performance. The sensitivity (recall) metric captures the model’s ability to successfully predict occurrences of the positive class (no-show), being one of the most important for performance assessment, as it directly impacts the reduction of false negatives (i.e., no-show cases predicted as show) [60]. AUC evaluates overall model discrimination ability across various thresholds, especially beneficial in unbalanced datasets without being biased by class imbalances.

**Fig. 2:**
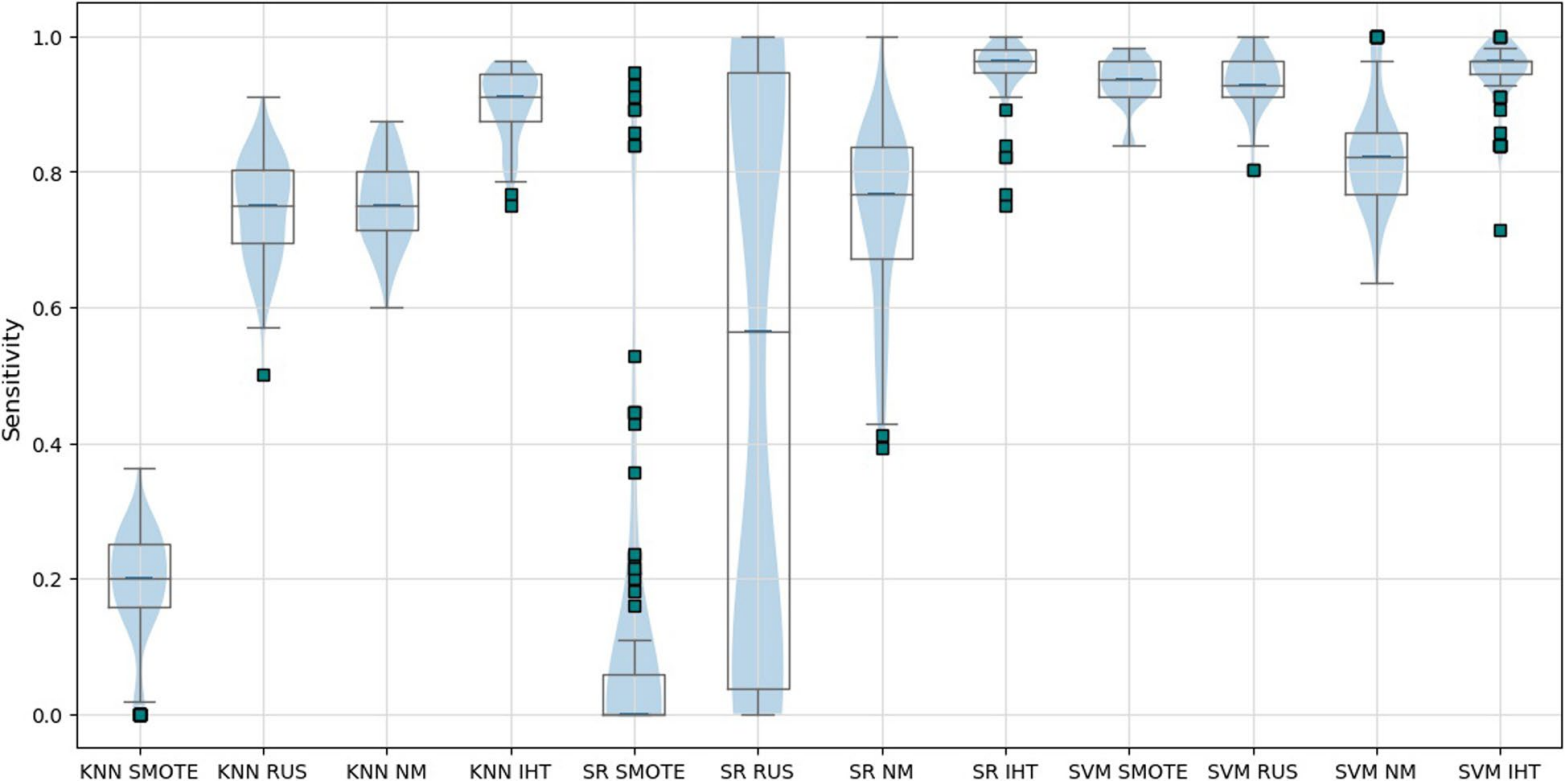
Boxplot of sensitivity results in the validation set for all prediction models

In the test set (Table 1), sensitivity and AUC values resulted larger than 0.9 for all combinations of classification algorithms with the IHT resampling technique, confirming its suitability for highly imbalanced datasets. In the validation set (Table 2), combining IHT with KNN and SR led to the best results in four of the metrics. The combination of SR and IHT yielded the best sensitivity score (0.9537) and AUC value (0.6175). However, it is notable that other metrics such as specificity, accuracy, NPV, PPV and F1_score are significantly impacted by the large class imbalance in the validation set, where 93.35% of observations belong to the ‘show’ class. It is noteworthy that the good sensitivity and AUC performance observed in the test set for IHT/KNN and IHT/SR combinations was maintained in the validation set. A boxplot of the sensitivity metric is presented to verify the stability of prediction models in the validation set considering cases correctly classified as no-shows (Fig. 2).

Some models are remarkably more stable than others, e.g., KNN/IHT, SR/IHT, SVM/IHT. SVM/IHT displayed the lowest overall interquartile dispersion, although the median line (positioned over the third quartile) reveals a negatively asymmetric data distribution. SR/IHT sensitivity results show high stability due to data symmetry and low variability, although presenting a few outliers (those positioned below the first quartile can negatively bias the results, but that was not the case with the model).

**Fig. 3:**
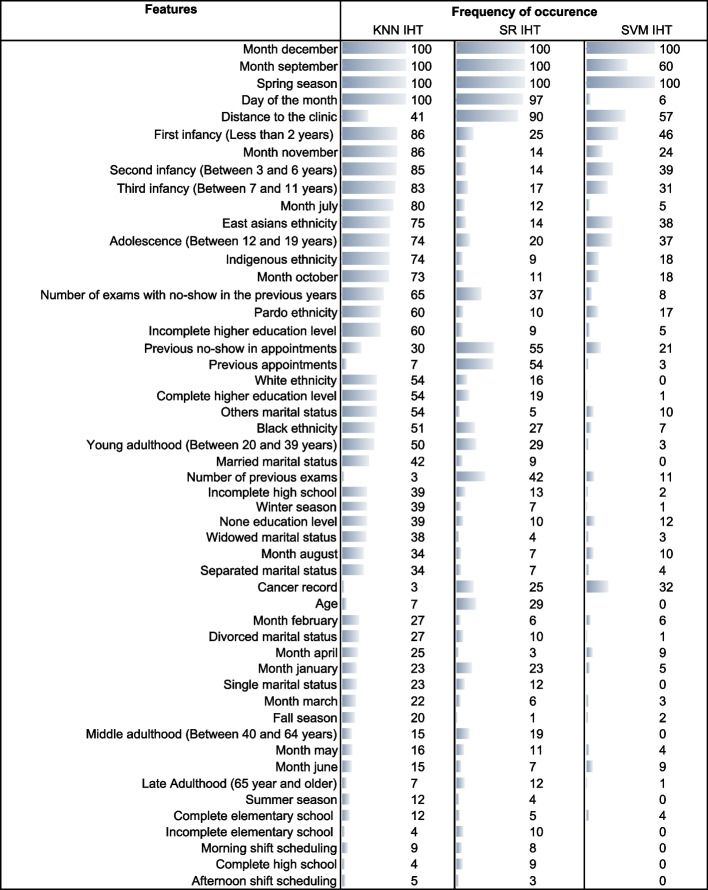
Features selected by top models, occurrence frequency in 100 test set replicates

Figure 3 lists the most frequently selected features from the test set for combinations of SR, KNN, and SVM classification algorithms with the IHT resampling technique. The top selected features will be discussed in “Results” section, considering the existing literature.

Applying the framework steps in Fig. 1 to Dataset 2 led to the results reported in Tables 3 (test set) and 4 (validation set). The presentation follows the same structure used in Dataset 1. The best performance was obtained using the prediction model combining SR and IHT on the test set, except for specificity and PPV metrics.

**Table 3:** Average predictive performance and standard deviations obtained from 100 replicates of dataset 2’s test portion

**Resampling technique**	**Classification algorithm**	**Performance Metrics – mean (SD)**						
		**AUC**	**Sensitivity**	**Specificity**	**NPV**	**PPV**	**F1_score**	**Accuracy**
SMOTE	KNN	0.8096 (0.072)	0.7630 (0.173)	0.8562 (0.079)	0.8010 (0.093)	0.8500 (0.059)	0.7886 (0.114)	0.8096 (0.071)
	SVM	0.7842 (0.040)	0.8234 (0.055)	0.7449 (0.085)	0.8105 (0.040)	0.7686 (0.057)	0.7925 (0.036)	0.7842 (0.040)
	SR	0.7375 (0.055)	0.6709 (0.177)	0.8041 (0.184)	0.7359 (0.106)	0.8123 (0.106)	0.7097 (0.076)	0.7375 (0.055)
RUS	KNN	0.6938 (0.050)	0.6528 (0.095)	0.7347 (0.040)	0.6841 (0.060)	0.7092 (0.044)	0.6776 (0.067)	0.6938 (0.050)
	SVM	0.6811 (0.051)	0.6031 (0.105)	0.7590 (0.041)	0.6619 (0.057)	0.7123 (0.044)	0.6497 (0.076)	0.6811 (0.051)
	SR	0.7187 (0.072)	0.7144 (0.232)	0.7231 (0.208)	0.7632 (0.131)	0.7600 (0.116)	0.6993 (0.121)	0.7187 (0.072)
NM	KNN	0.7543 (0.058)	0.6738 (0.124)	0.8349 (0.058)	0.7278 (0.076)	0.8053 (0.057)	0.7273 (0.082)	0.7543 (0.058)
	SVM	0.7703 (0.047)	0.6827 (0.095)	0.8580 (0.069)	0.7350 (0.058)	0.8337 (0.064)	0.7456 (0.060)	0.7703 (0.047)
	SR	0.7509 (0.084)	0.6551 (0.188)	0.8468 (0.127)	0.7302 (0.100)	0.8201 (0.122)	0.7091 (0.141)	0.7509 (0.084)
IHT	KNN	0.9399 (0.021)	0.9396 (0.038)	0.9401 (0.041)	0.9512 (0.030)	0.9307 (0.041)	0.9339 (0.023)	0.9398 (0.022)
	SVM	0.9321 (0.037)	0.9137 ( 0.070)	**0.9505 (0.037)**	0.9340 (0.048)	**0.9397 (0.040)**	0.9246 (0.043)	0.9340 (0.035)
	SR	**0.9429 (0.048)**	**0.9425 (0.094)**	0.9433 (0.046)	**0.9570 (0.057)**	0.9350 (0.047)	**0.9349 (0.065)**	**0.9429 (0.045)**

**Table 4:** Average predictive performance and standard deviations obtained from 100 replicates of dataset 2’s validation portion

**Resampling technique**	**Classification algorithm**	**Performance Metrics – mean (SD)**						
		**AUC**	**Sensitivity**	**Specificity**	**NPV**	**PPV**	**F1_score**	**Accuracy**
SMOTE	KNN	0.7105 (0.068)	0.5346 (0.171)	**0.8864 (0.086)**	0.8928 (0.032)	**0.5592 (0.115)**	0.5246 (0.107)	**0.8194 (0.055)**
	SVM	0.7400 (0.056)	0.6958 (0.161)	0.7841 (0.097)	0.9202 (0.033)	0.4565 (0.101)	**0.5327 (0.073)**	0.7673 (0.060)
	SR	0.7140 (0.070)	0.6004 (0.226)	0.8277 (0.159)	0.9058 (0.043)	0.5409 (0.165)	0.5161 (0.103)	0.7844 (0.098)
RUS	KNN	0.7317 (0.046)	0.7319 (0.100)	0.7315 (0.077)	0.9217 (0.022)	0.4019 (0.073)	0.5116 (0.055)	0.7316 (0.055)
	SVM	0.7057 (0.051)	0.6487 (0.117)	0.7628 (0.089)	0.9039 (0.025)	0.4045 (0.073)	0.4908 (0.068)	0.7411 (0.062)
	SR	0.7163 (0.072)	0.7103 (0.258)	0.7222 (0.217)	0.9277 (0.049)	0.4661 (0.162)	0.4976 (0.094)	0.7199 (0.137)
NM	KNN	0.6459 (0.076)	0.7811 (0.135)	0.5107 (0.224)	0.9160 (0.035)	0.2935 (0.068)	0.4175 (0.070)	0.5622 (0.163)
	SVM	0.6155 (0.061)	0.8288 (0.106)	0.4021 (0.169)	0.9142 (0.034)	0.2532 (0.040)	0.3845 (0.046)	0.4833 (0.124)
	SR	0.6900 (0.078)	0.7280 (0.168)	0.6521 (0.206)	0.8690 (0.203)	0.3660 (0.104)	0.4659 (0.088)	0.6665 (0.148)
IHT	KNN	0.7544 (0.033)	0.9418 (0.035)	0.5670 (0.067)	0.9768 (0.013)	0.3418 (0.035)	0.5004 (0.038)	0.6384 (0.053)
	SVM	0.7684 (0.048)	0.9120 (0.079)	0.6248 (0.069)	0.9688 (0.025)	0.3680 (0.048)	0.5226 (0.054)	0.6795 (0.056)
	SR	**0.7734 (0.038)**	**0.9434 (0.087)**	0.6033 (0.077)	**0.9802 (0.020)**	0.3661 (0.054)	0.5214 (0.045)	0.6681 (0.055)

In the validation set, the KNN/SMOTE yielded the best specificity, PPV, and accuracy results. However, its sensitivity (0.5346) suggests that this model combination may not be the most suitable, considering the high cost of a false negative for healthcare centers. On the other hand, the SR/IHT combination resulted in the best sensitivity (0.9434) and AUC (0.7734), indicating that this combination might be more favorable, especially considering the importance of minimizing false negatives.

**Fig. 4:**
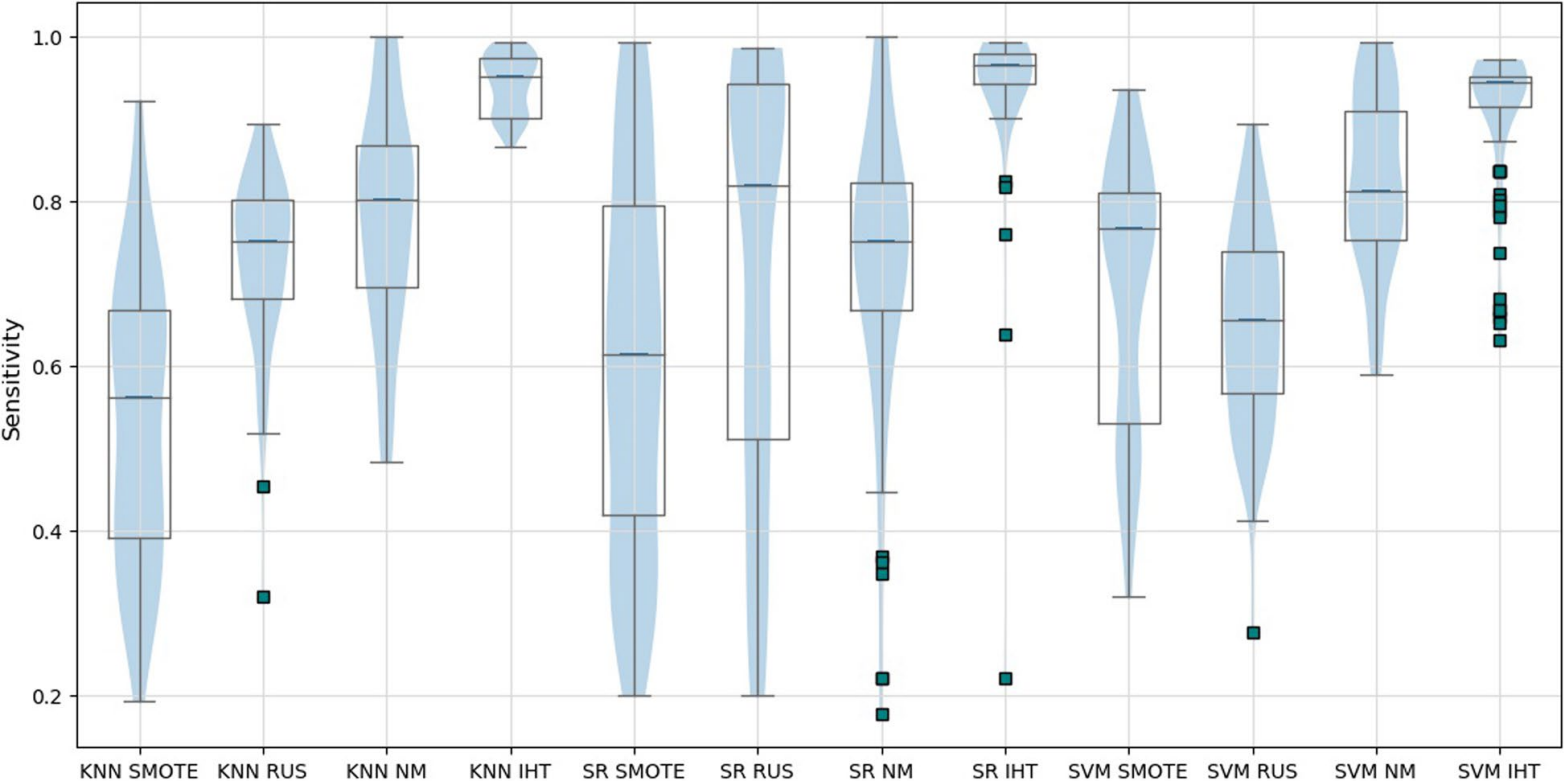
Boxplot of sensitivity results in the validation set for all prediction models

The stability of prediction models in the validation set considering cases correctly classified as no-show is presented in the boxplot of the sensitivity metric (Fig. 4). Classification algorithms KNN, SR, and SVM, in combination with the IHT technique, presented the smallest dispersion. Model SVM/IHT presents a negatively asymmetric data distribution (median positioned close to the third quartile), indicating that most data points are positioned above the median. On the other hand, models SR/IHT and KNN/IHT are nearly symmetric, indicating stability.

**Fig. 5:**
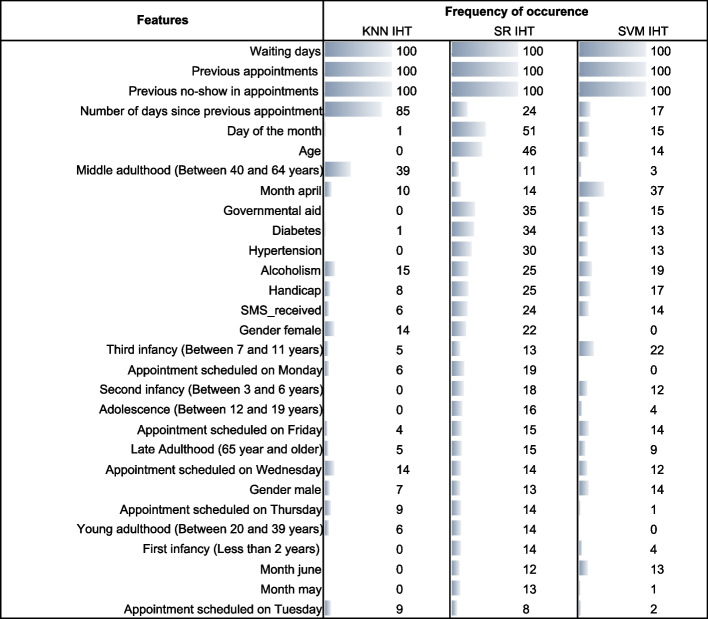
Features selected by top models, occurrence frequency in 100 test set replicates

Figure 5 displays the most frequently selected features from the test set for combinations of KNN, SR, and SVM classification algorithms with the IHT resampling technique. The top selected features will be discussed in “Results” section.

## Discussion

Our results are now discussed considering the no-show prediction literature considering important aspects of our analytical framework: dataset stratification and folding for model performance assessment, resampling for dataset balancing, the set of features available for analysis and those significant for no-show prediction and practical implications.

### Cross-validation strategies and stratification

Ideally, prediction models are generalizable, i.e., when classifying observations not previously used for training and testing, they should yield performance like that verified in testing sets. We conducted a comprehensive review of 62 studies on no-shows, as detailed in Table S1. To obtain generalizable no-show models, cross-validation strategies are reported in the literature [28, 37, 38, 40, 48, 57–59, 69, 70], mostly the random division of datasets into calibration and validation portions, followed by cross-validation of the calibration portion in which train and test subsets are divided into 5 or 10-folds. In those studies, the validation set remained the same during model training, and a single performance result was obtained for the best model, i.e., no measure of performance dispersion became available. Aladeemy et al. [31] performed the cross-validation as the ten previous studies but repeated the process 3 times, on the validation portion obtaining more than one validation sample. Krishnan and Sangar [21] were the only study reporting foldings of the validation set (4 × [5-folds]) using the entire dataset. However, foldings were obtained randomly, which may lead to different class frequencies in the calibration and validation sets.

Some authors [3, 26, 29, 34, 35, 49, 71–73] divided the complete dataset into train and validation portions using 10-fold partitions. However, analyzing the results in “Datasets” section, it is safe to assume that only 10 simulations may be insufficient to capture the generalizability and stability of the models obtained in the train portion. The SR/RUS model, for example, presented results with variability ranging from 0 to 100% over the 100 replicates, as shown in the sensitivity boxplots in Figs. 2 and 4. If only 10 validation runs were considered, results could be biased for better or worse. Contrastingly, employing cross-validation across two stages enables 100 simulations across distinct folds, curbing repetitions and strengthening result robustness. This method substantially tackles generalization and stability issues in predictive models, especially amidst substantial class frequency variations. Thus, our strategy promises a significant advancement in evaluating predictive models, ensuring their reliability and adaptability for real-world applications.

While this technique demands added computational resources due to more numerous simulations, leveraging technological advancements and parallel processing capabilities can streamline this phase. Moreover, bolstering model generalization and stability holds importance, particularly in sensitive fields like medicine, finance, and complex event forecasting, where unreliable results could lead to adverse consequences. The method’s adaptability across diverse datasets and contexts further underscores its efficacy, paving the way for versatile and dependable deployment in real-world scenarios.

Finally, authors [3, 28, 29, 37, 38, 40, 57, 69] reported the stratified partitioning of datasets to ensure that classes in train, test and validation portions displayed the same proportions as the entire dataset. However, no treatment of class imbalance was reported. Implementing proportional class sampling (Stratification by class) when splitting the dataset into cross-validation folds, such that the incidence of each class in the folds reflects that of the entire dataset, also leads to more reliable results [37, 38, 40].

### Resampling techniques

The magnitude of imbalance between classes may undermine the predictive power of machine learning algorithms, as the learning stage becomes difficult and favors the adoption of naive approaches to minimize the loss function during the classification process, leading to models that cannot successfully differentiate between classes [21]. The problem escalates as the dataset imbalance increases, with prediction models that generate biased results (i.e., false alarms) that are not usable in practice [21, 28], since the model will tend to classify new cases as belonging to the majority class [21]. Resampling techniques are an alternative to address this problem.

In the studies surveyed in Table S1, the no-show rate was lower than the attendance rate of patients in most medical specialties, except for Alshammari et al. [74] and Bhavsar et al. [48]. Resampling techniques are an alternative for dealing with imbalanced datasets [21]. They are applied before the algorithm learning process. Examples are minority class oversampling, majority class undersampling, or combinations of both [35]. Resampling techniques perform differently according to the dataset at hand. The best technique is the one that captures the disparities between classes, resulting in the best prediction performance [35].

To overcome class imbalance, resampling techniques (mostly undersampling) were applied by eleven of the authors listed in Table S1 [21, 26, 28, 29, 31, 32, 34, 35, 58, 59, 73]. All eleven studies that used resampling techniques displayed sensitivity results of at least 64%. Of the remaining studies that did not apply resampling, 18 reported sensitivity results: five of them were between 20 and 55% [2, 41, 45–47], reflecting a low probability of correctly identifying no-show cases; other remaining eleven studies reported sensitivity results larger than 64% [1, 20, 23, 27, 39, 69, 74–78], although calculated on a single fold of the validation set, which is likely to yield biased results.

In our proposed framework, we recommend using resampling techniques to minimize imbalance in no-show datasets, followed by controlled stratification and cross-validation to obtain generalizable models. The following two other studies adopted a similar strategy. In AlMuhaideb et al. [29], the complete dataset was divided using a 10-fold cross-validation, but the number of folds used for calibration and validation was not reported. Random undersampling was the resampling technique adopted. As mentioned earlier, only 10 simulations may be insufficient to produce stable and generalizable models. Nasir et al. [28] randomly divided the dataset into calibration (20%) and validation (80%) portions. A 5-fold cross-validation was applied only in the calibration set. The 20%/80% splitting of the dataset may not be the most adequate: according to Srinivas and Salah [57], models obtained using larger train sets are more generalizable. In addition, validation was performed on a single fold.

Although not reporting stratification strategies, the combination of resampling and cross-validation techniques was also applied in 9 other studies [21, 26, 31, 32, 34, 35, 58, 59, 73]. Considering those in which no-show was the minority class and AUC was larger than 0.60, the best sensitivity results (0.89) were obtained by Starnes et al. [76] and Joseph et al. [78]. In our study, the combination of IHT and SR yielded sensitivity results greater than 0.94 in the two analyzed datasets, i.e., representing the most favorable outcomes reported in the literature, to the best of our knowledge. It is essential to note that our study, while showcasing this absolute advantage, did not perform specific statistical analyses to confirm significant differences. The presence of overlap and imbalance among classes complicates classification. We believe that the outstanding performance of IHT lies in its ability to identify these challenging instances, allowing for their removal during machine learning model training. This results in significant improvements in the separation between classes, directly impacting classification results.

### Significant predictors

We now analyze the set of predictors most frequently selected in modeling the two datasets in our study. The variable present in both datasets and most frequently selected was ‘day of the month’. Variables ‘month of the year’ and ‘age’, frequently selected when using dataset 1, were also selected when using dataset 2, but with lower frequency. On the other hand, variables ‘waiting days’, ‘previous appointments’, and ‘percentage of previous no-shows’, present in both datasets, were selected more frequently only when using dataset 2. Datasets reflect specific cases and, therefore, present different information. For example, frequently selected variables ‘season of the year’, ‘distance to the clinic’, and ‘number of exams with no-show in the previous year’ were available in dataset 1 but not in dataset 2; similarly, the frequently selected variable ‘number of days since previous appointment’ was only available in dataset 2. Prediction quality is dependent on the volume and diversity of the information available in the dataset [10, 26, 35]. As in our work, many authors [3, 5, 22, 26, 27, 33, 79] reported the non-availability of information as a limiting factor for accurate no-show predictions. Furthermore, most predictors displayed importance levels that varied depending on the classification algorithm being tested. According to Nasir et al. [28], that is due to the different processing strategies performed by the algorithms.

The most frequently retained variables found in our study were consistent with the results found in the literature. For example, age [1–8, 10, 18–20, 22–24, 26–29, 31, 34, 37, 38, 40, 45–48, 57, 75, 76, 80–86], day of the month [26, 33, 35, 40], month of the year [1–3, 7, 18, 26, 48, 80, 82], season of the year [6, 19, 22, 25, 33, 45], distance to the clinic [1–3, 5, 6, 8, 10, 18, 23–25, 30, 75, 81, 82], previous no-shows [1, 2, 4, 7, 10, 18, 20, 23, 26–28, 35, 47, 57, 80–82, 85, 87], previous appointments [1–3, 10, 19, 20, 40, 81], number of days since previous appointment [26, 28, 32, 33, 35, 40, 48] and waiting days [1, 26–28, 31, 32, 35, 37, 38, 40, 48, 49, 79, 80, 83] were predictors associated with no-show in previous studies. The predictor ‘number of exams with no-show in the previous year’, selected in our analysis, was not available in other datasets reported in the literature.

Although different variables were identified as significant predictors of no-show in our and previous studies, results are not always generalizable since no-show is a case-specific phenomenon affected by internal and external factors which may be exclusive to each medical service. For example, gender appears as a significant no-show predictor in the works of Mander et al. [5], who found a higher no-show rate in male individuals, and AlRowaili et al. [30], who found the opposite.

Despite not being entirely generalizable, studies that identify significant no-show predictors in each socioeconomic context might help managers devise compensating strategies to reduce its effects. For example, in Glover et al. [24], no-show was associated with transport barriers faced by low-income patients. To overcome that, patients were directed to more geographically accessible clinics for consultation, partnerships with public and private transport managers were created, and a free transportation program was proposed for the most vulnerable patients.

### Practical implications

The annual costs of CT scan examinations for the 557 (6.65%) no-show cases in Dataset 1 translated to an annual financial loss ranging between US$ 12,574.40 and US$ 21,149.18. Financial information for Dataset 2 is unavailable. In order to minimize the impacts of no-shows, patient reminders and overbooking emerge as strategies commonly discussed in the literature. Patient reminders and overbooking are strategies commonly presented in the literature to minimize the negative impacts of no-shows. Patient reminders, e.g., phone calls, text messages, and e-mails, are used to prevent patients from forgetting their appointments. Robotic auto calls are low-cost alternatives, although not as effective as resource-demanding personalized reminders [22]. Overbooking is a strategy in which more than one patient is scheduled for the same time slot. It potentially increases the system’s revenues by reducing idle times. However, it may also lead to problems such as system’s overcrowding and patients’ longer waiting times [3, 22, 81]. Our study has practical implications since knowledge of most likely no-show patients allows directing strategies such as patient reminders and overbooking to those patients, optimizing the use of resources.

## Conclusion

No-shows to medical appointments have negative impacts on healthcare systems and their clients. Using statistical methods to forecast no-shows allows managers to adopt more effective and proactive strategies to mitigate the problem. In this study, we propose an analytical framework for predicting no-shows, aiming to reduce bias in the predictive process and generate potentially generalizable results. Other objectives were to test methods not yet explored in the literature (SR and IHT) and to compare the performance of different combinations of classification algorithms and resampling techniques.

To the best of our knowledge, we are the first to propose using *z*-fold cross-validation twice in the modeling process (steps 2 and 4 of Fig. 1), resulting in 100 replicates of each prediction model tested. That allows a more comprehensive assessment of performance metrics by determining their centrality and dispersion statistics and minimizes the possibility of bias in the composition of the calibration and validation sets. We also innovate by proposing the use of SR as a classification algorithm and IHT as a resampling technique, both of which presented superior performances compared to other techniques, particularly IHT, which excelled when combined with all classification algorithms and led to low variability in performance metrics results.

As in other studies reported in the literature, prediction models considered only the predictors available in the datasets analyzed, which reflect information from appointment scheduling systems. Therefore, candidate predictors were not necessarily inserted in the datasets with the objective of describing the no-show phenomenon. In future studies, we propose designing datasets tailored for no-show prediction using qualitative expert inputs. Considering the superior performance displayed by combinations of the IHT technique and classification algorithms, we also propose expanding the application of such prediction models to other highly imbalanced datasets.

### Supplementary Information

**Additional file 1: Table S1.** No-show modeling approaches in the literature.

## Data Availability

Dataset 1 that support the findings of this study are available from the corresponding author, upon reasonable request. Dataset 2 is publicly available and was acquired from the Kaggle platform https://www.kaggle.com/datasets/joniarroba/noshowappointments.
