# Supplementary material for: Decision analysis framework for predicting no-shows to appointments using machine learning algorithms
Source: BMC Health Serv Res. 2024 Jan 5;24:37. doi: 10.1186/s12913-023-10418-6 (PMC10770919; doi:10.1186/s12913-023-10418-6)
Supplement: Supplementary file 1 — Additional file 1: Table S1. No-show modeling approaches in the literature. [file 12913_2023_10418_MOESM1_ESM.docx]

**Table S1**

No-show modeling approaches in the literature

| **Authors (year)^(ID)^** | **No-show rate** | **Best performing resampling techniques** | **Dataset % division** | **Cross-validation [Z-folds]** | **Stratification by class** | **Best performing classification algorithms** | **Feature Selection** | **Sector** |
| --- | --- | --- | --- | --- | --- | --- | --- | --- |
|  |  |  | [train, test, **valid]** |  |  |  |  |  |
| Dunstan et al. (2023) | 20.40 | - | - | [10-folds] in all dataset | - | Balanced Bagging Classifier | Filter | Different Specialties in a pediatric hospital |
| Alshammari, Daghistani and Alshammari (2020) | 88.89 | - | [70, 0, 30] | - | - | Artificial Neural Network (ANN) | - | Ambulatory |
| Salah and Srinivas (2022) | - |  |  | [10-folds] only in train set |  |  | - | Endocrinology |
| Liu et al. (2022) | 20.00 | - | - | [10-folds] in all dataset |  |  | Filter | Pediatrics |
| Simsek, Tiahrt and Dag (2020) | 20.93 | Random Under Sampling |  |  |  |  | Wrapper | - |
| Daghistani et al. (2020) | 26.71 | - | [70, 0, 30] | - | -  Yes | Gradient Boosting (GB) | Filter | Ambulatory |
| Lekham et al. (2020) | 26.10 |  |  | [10-folds] only in train set |  |  |  | Different Specialties |
| Ferreira and Vasconcelos (2022) | - | SMOTE | - | [10-folds] in all dataset | - |  |  | - |
| Elvira et al. (2018) | 10.60 | - | [60, 20, 20] | - | - |  | - | Primary Care |
| Alshammari et al. (2022) | 44.56 |  | [80, 0, 20] |  |  |  |  | Pediatrics |
| Joseph et al. (2022) | 20.93 |  |  |  |  |  |  | - |
| Chen et al. (2020) | 15.40 | - | [75, 0, 25] | [5-folds] only in train set | Yes | XGBoost | Embedded | Pediatric Ophthalmology |
| Cui and Finkelstein (2022) | 2.00 | Random Under Sampling | [70, 0, 30] | [3-folds] only in train set | - |  | - | Primary Care |
| Chong et al. (2020) | 17.40 |  | [80, 0, 20] | [10-folds] in train and test |  |  |  | Magnetic Resonance |
| Incze et al. (2021) | 17.00 | - | [80, 10, 10] | [not inf. -fold] only in train set | Yes | LightGBM | - | Ambulatory |
| Srinivas and Salah (2021) | 18.00 | - | [75, 0, 25] | [10-folds] only in train set | Yes | Stochastic Gradient Boosting | - | Cardiology |
| Simsek et al. (2021) | 20.93 | Random Under Sampling | - | [10-folds] in all dataset | - | Bayesian Belief Network (BBN) | Wrapper | - |
| Topuz et al. (2017) | 27.3 | - | - | [10-folds] in all dataset | Yes |  | Embedded | Pediatrics |
| Devasahay, Karpagam and Ma (2017) | 18.59 | - | Not inf. | - | - | Decision Tree (DT) | Filter | Different Specialties |
| Valero-Bover et al. (2022) | 20.00 |  | [75, 0, 25] | [5-folds] only in train set | Yes |  |  | dermatology and pneumology |
| Lotfi and Torres (2014) | 15.00 | - | [55, 0, 45] | - | - |  | Embedded | Physiotherapy |
| AlMuhaideb et al. (2019) | 11.30 | Random Under Sampling | - | [10-folds] in all dataset | Yes |  |  | Different Specialties |
| Aladeemy et al. (2020) | 18.58 |  | [70, 0, 30] | 3 * [5-folds] only in train set | - |  | Wrapper | Primary Care |
| Krishnan and Sangar (2021) | 20.00 | Condensed Nearest Neighbor (CNN) | - | 4 * [5-folds] in all dataset | - |  | - | - |
| Mohammadi et al. (2018) | 17.00 | - | 10 * [70, 0, 30] |  |  | Naïve Bayes | Filter | Primary Care |
| Nasir et al. (2020) | - | Compared techniques, but the best performances were without using | [20, 0, 80] | [5-folds] only in train set | Yes | Random Forest (RF) | - | - |
| Taheri-Shirazi et al. (2023) | 3.61 | - | [75, 0, 25] | - | - |  | Filter | Magnetic Resonance |
| Abushaaban and Agaoglu (2023) | 20.93 | Random Under-sampling. | [80, 0, 20] | [10-folds] only in train set | - |  | - | - |
| Ahmadi et al. (2019) | 23.00 | Hybrid of an Over Sampling Under Sampling | [70, 0, 30] | [not inf. -fold] set not inf. | - |  | Wrapper | Neurology |
| Davis et al. (2020) | 16.30 | - | [100, 0, 0] | - | - | Generalized Linear Mixed Effects Model (GLMM) | Embedded | Psychological Therapy |

| **Authors (year)^(ID)^** | **No-show rate** | **Best performing resampling techniques** | **Dataset % division** | **Cross-validation [Z-folds]** | **Stratification by class** | **Best performing classification algorithms** | **Feature Selection** | **Sector** |
| --- | --- | --- | --- | --- | --- | --- | --- | --- |
|  |  |  | **[train, test, valid]** |  |  |  |  |  |
| Lenzi, Bem and Stein (2019) | 13.00 | - | [50, 0, 50] | - | - | Mixed Effects Logistic Regression (MELR) | Wrapper | Primary Care |
| Li et al. (2019) | 18.00 | - | [80, 0, 20] | - | - |  | - | Primary Care |
| Kurasawa et al. (2016) | 5.80 | - | - | [10-folds] in all dataset | - | Logistic Regression using Ridge | Embedded | Diabetes Diagnosis |
| Bhavsar et al. (2021) | 73.10 | - | [75, 0, 25] | [10-folds] only in train set | - | Logistic Regression using Lasso | Embedded | Physiotherapy |
| Lin et al. (2019) | 18.00 | - | [80, 0, 20] | - |  |  |  | Different Specialties |
| Ding et al. (2018) | 13.00 - 32.00 | - | [67, 0, 33] |  |  |  |  |  |
| Mieloszyk et al. (2019)^(1)^ ;Rosenbaum et al. (2018)^(2)^; Blaehr et al. (2016)^(3)^; Peng et al. (2014)^(4)^ | From 2.60 to 17.01 | - | [100, 0, 0] | - | - | Logistic regression (LR) | - | Radiology^(1, 2, 3)^; Endoscopy^(4)^ |
| Gromisch et al. (2020)^(1)^; Starnes et al. (2019)^(2)^; Parente et al. (2018)^(3)^ | From 4.74 to 30.80 | - | - | - | - | Logistic regression (LR) | Filter | Chronic Care ^(1)^; Primary Care^(2)^; Radiology^(3)^ |
| Chua and Chow (2019) | 28.60 |  | [15, 35, 50] |  |  |  |  | Ambulatory |
| Dantas et al. (2018)^(1)^; Torres et al. (2015)^(2)^ | From 45.30 to 21.90 |  | [70, 0, 30] |  |  |  |  | Bariatric ^(1)^; Pediatrics ^(2)^ |
| Mander et al. (2018); Lu et al. (2017); AlRowaili et al. (2016) | From 5.40 to 34.80 |  | [100, 0, 0] |  |  |  |  | Radiology |
| Odonkor et al. (2017) | 24.60 |  | - | 3 × 10^4^ random iterations |  |  |  | Pain Clinic |
| Huang and Hanauer (2014) | 11.20 |  | [80, 0, 20] |  |  |  |  | Primary Care |
| Huang and Hanauer (2016)^(1)^; Giunta et al. (2013)^(2)^ | From 22.60 to 17.00 |  | [67, 0, 33] |  |  |  |  | Pediatrics ^(1)^; Ambulatory ^(2)^ |
| Blumenthal et al., (2015) | 13.69 |  | [78, 0, 22] |  |  |  |  | Chronic Care |
| Daye et al. (2018); Glover et al. (2017); Harvey et al. (2017) | From 4.50 to 6.50 |  | [100, 0, 0] |  |  | Logistic regression (LR) | Wrapper | Radiology |
| Milicevic et al. (2020) | 19.61 |  | [70, 0, 30] |  |  |  |  | Mental Health |
| Goffman et al. (2017) | 13.87 |  | [63, 0, 38] |  |  |  |  | Ambulatory |
| Daggy et al. (2010) | 15.20 |  | [67, 0, 33] |  |  |  |  |  |
